# Die Evolution der Konzeption der Neurosen in den Lehrbüchern Emil Kraepelins

**DOI:** 10.1007/s00115-025-01821-x

**Published:** 2025-03-19

**Authors:** Clara Fuhrmann, Holger Steinberg

**Affiliations:** Forschungsstelle für die Geschichte der Psychiatrie, Klinik und Poliklinik für Psychiatrie und Psychotherapie, Medizinische Fakultät der Universität Leipzig, Semmelweisstraße 10, 04103 Leipzig, Deutschland

**Keywords:** Neurosen, Psychogene Erkrankungen, Nosologie psychischer Erkrankungen, Psychiatriegeschichte, Emil Kraepelin, Neuroses, Psychogenic diseases, Nosology of mental illnesses, History of psychiatry, Emil Kraepelin

## Abstract

**Hintergrund:**

Der deutsche Psychiater Emil Kraepelin (1856–1926) entwarf eine Nosologie psychischer Erkrankungen, die in ihren Grundzügen, insbesondere in Gestalt der Abgrenzung der affektiven und schizophrenen Formenkreise, bis heute Bestand hat. Wenig ist dagegen über seine Arbeit zu den Neurosen bekannt.

**Fragestellung:**

Welche Entwicklung durchlief Kraepelins Neurosenkonzeption vor allem bezüglich ihrer ätiologischen Theorien, Grundcharakteristika, Symptomkomplexe und damit auch ein- oder ausgeschlossenen Störungen? Welche Einflussfaktoren spielten dabei jeweils eine entscheidende Rolle?

**Material und Methoden:**

Alle neurosenspezifischen Kapitel der 1. bis 8. Auflage des Psychiatrielehrbuches Kraepelins, die zwischen 1883 und 1915 erschienen, wurden analysiert, verglichen und auf Veränderungen sowie deren Veränderungsmotivationen hin überprüft.

**Ergebnisse:**

Die in der 2. Auflage etablierte Neurosenkonzeption wird in ihren Grundzügen bis zur 6. Auflage beibehalten. In der 7. Auflage bricht Kraepelin aufgrund mangelnder Trennschärfe mit seiner ursprünglichen Konzeption und behält lediglich eine Untergruppe bei: die „psychogenen Neurosen“. Diese wird in der 8. Auflage unter dem Namen der „psychogenen Erkrankungen“ fortgeführt und durch neue Störungsbilder erweitert.

**Diskussion:**

Für die Entwicklung der Neurosenkonzeption Kraepelins lassen sich sowohl gesellschaftliche als auch fachlich nervenärztliche Einflussfaktoren identifizieren.

## Abstract (en)

**Background:**

The German psychiatrist Emil Kraepelin (1856–1926) developed a nosology of mental illnesses, the main features of which are still valid today, particularly in the form of the differentiation between the affective and schizophrenic forms. However, little is known about his work on neuroses.

**Objective:**

What development did Kraepelin’s concept of neurosis undergo, especially with respect to its etiological theories, symptom complexes, basic characteristics and thus also included or excluded disorders?

Which influencing factors played a decisive role in each case?

**Material and methods:**

All neurosis-specific chapters of the 1st to 8th editions of Kraepelin’s psychiatry textbook published between 1883 and 1915 were analyzed, compared and examined for changes and their motivations for change.

**Results:**

The neurosis concept established in the 2nd edition is retained in its basic features until the 6th edition. In the 7th edition Kraepelin breaks with his original conception due to a lack of selectivity and only retains one subgroup: the “psychogenic neuroses”. This was continued in the 8th edition under the name of “psychogenic disorders” and expanded to include new disorders.

**Conclusion:**

Both social and professional neurological influencing factors can be identified for the development of Kraepelin’s concept of neuroses.

## Hintergrund

An der Schwelle zum 20. Jahrhundert entwarf der deutsche Psychiater Emil Kraepelin (1856–1926) die erste, schnell weithin akzeptierte Nosologie psychischer, sich auf den Ebenen des Denkens, Fühlens oder Verhaltens manifestierender, Erkrankungen. Die Grundzüge dieser Krankheitslehre schlagen sich noch bis heute in unseren diagnostischen Manualen nieder. So geht auf ihn die fundamentale Dichotomie der Erkrankungen des schizophrenen und affektiven Formenkreises zurück, welche er in der 6. Auflage seines Psychiatrielehrbuches etablierte [11, 18]. Wenig ist dagegen über seine Arbeit zu den Neurosen bekannt. Der Begriff der Neurose wurde 1776 von William Cullen (1710–1790) geprägt und bezeichnete Erkrankungen, deren unmittelbare Ursache in einer Beeinträchtigung der Nervenfunktion bestand. Im 19. Jahrhundert setzte im Kontext der aufkommenden pathologischen Anatomie ein Spaltungsprozess der Neurosen Cullens ein. Philippe Pinel (1745–1826) war 1789 der erste, der nur noch diejenigen Erkrankungen als Neurosen verstand, denen kein nachweisbares organisches Korrelat zugrunde lag – eine Auffassung die sich im 19. Jahrhundert zunehmend durchsetzte. Neurosen galten zwar weiterhin als Erkrankungen mit körperlicher Grundlage, diese wurde jedoch als unentdeckt oder gänzlich unentdeckbar aufgefasst [8, 32], bis Sigmund Freud (1856–1939) sie zu psychogenen, aus psychischen Vorgängen entstehenden, Erkrankungen erklärte [7, 37].

Die vorliegende Arbeit widmet sich der Frage, welche ätiologischen Konzepte, Grundcharakteristika, dominierenden Symptomkomplexe und damit auch ein- oder ausgeschlossenen spezifischen Störungen Kraepelin im Verlauf seines Schaffens mit diesem Begriff verband und welche Veränderungsmotivationen sich hinter den jeweiligen Entwicklungsstufen verbergen mögen. Zu einer besseren Einordnung der historischen Krankheitsentitäten werden zusätzlich moderne Nachfolger der initialen Neurosen Kraepelins diskutiert.

## Methoden

Im Laufe seiner wissenschaftlichen Karriere nutzte Emil Kraepelin die regelmäßigen Neuauflagen seines Psychiatrielehrbuches, um im Sinne einer heuristischen Selbstbefragung seine jeweils aktuellen Auffassungen zum Fach Psychiatrie, aber vor allem zu Nosologie, Symptomatologie, Ätiologie und Therapie der einzelnen psychischen Erkrankungen darzulegen. Insofern eignet sich das systematische Heranziehen der einzelnen Auflagen zur Gewinnung einer chronologischen Übersicht über die Evolution sowohl seiner allgemeinen Gruppierung von Krankheiten als auch einzelner Entitäten.

**Abb. 1:**
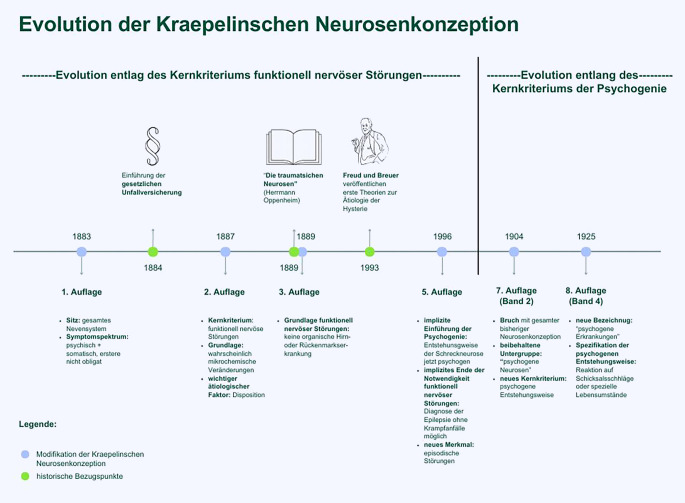
Evolution der Neurosenkonzeption Kraepelins

Die vorliegende Arbeit konzentriert sich auf die zwischen 1883 und 1915 erschienene 1. bis 8. Auflage, da Kraepelin während der Arbeit an seiner 9. Lehrbuchauflage verstarb und ihre Niederschrift größtenteils durch seinen Schüler Johannes Lange erfolgte [14, 28]. Jeweils wurde pro Auflage folgendes Textmaterial exzerpiert, inhaltlich analysiert und abschließend jeweils mit den Analysen der vorherigen Auflagen verglichen: erstens ein kurzer, neurosenspezifischer Abschnitt im ursachenzentrierten Teil des Lehrbuches und zweitens alle Kapitel, die sich einzelnen neurotischen Erkrankungen widmen, mitsamt ihrer gemeinsamen Einleitung. Die signifikanten, hierbei identifizierten Ausgangs- und Veränderungspunkte werden dargestellt und auf mögliche Veränderungsmotivationen hin überprüft. Abb. 1 stellt die wesentlichsten Entwicklungen zusätzlich visuell dar.

## Erste und zweite Auflage

Die 1. Auflage, das lediglich 384-seitige, taschenbuchartige „Compendium der Psychiatrie“, das auf nur wenigen Jahren als klinisch-psychiatrischer Assistent des Autors beruht, gibt folgende krankheitsübergreifende Neurosencharakteristika preis: Ihr Sitz sei im gesamten Nervensystem und nicht primär im Großhirn zu lokalisieren. Zu ihrem Symptomspektrum, dessen Komponenten für gewöhnlich auf die „gleiche allgemeine centrale Ursache“ zurückgeführt werden könnten, würden sowohl körperliche als auch psychische Krankheitszeichen gehören, wobei den letzteren kein explizit obligatorischer Charakter zukomme [19, S. 22].

Die 2.,1887 erschienene Auflage beinhaltet neben drei krankheitsspezifischen Kapiteln zu Hysterie, Epilepsie und Neurasthenie auch drei weitere grundlegende Eigenschaften der Neurosen: Erstens würden sie stets mit „functionell nervösen Störungen“ einhergehen, die sich phänomenologisch in Kopfschmerzen, geringer körperlicher Belastbarkeit, druckschmerzhaften Nervenaustrittsstellen, Schwindel, Seh- und Sprechstörungen, Muskelzuckungen, Parästhesien, Ausdrucksbewegungen, Lähmungen, Krämpfen, Appetitlosigkeit und Erbrechen äußern würden [20, S. 374–376, S. 393]. Im Kontext der Neurosen bleibt der Autor eine tiefergehende Definition dieses Begriffs zunächst schuldig, er attestiert jedoch der Mehrzahl aller psychischen Störungen – damit wahrscheinlich auch den Neurosen – ein körperliches Korrelat in Form „functioneller“ Veränderungen, „die der anatomischen Forschung höchstens mithilfe der Mikrochemie einmal annähernd zugänglich werden“ könnten [20, S. 210]. Dies fügt sich in die damals vorherrschende Meinung ein, neurotische Erkrankungen besäßen eine „körperlicher Basis“, welche den Methoden der pathologischen Anatomie aufgrund ihrer – wahrscheinlich molekularen – Struktur nicht zugänglich sei [9, S. 26–27].

Zweitens würde ein Großteil der Neurosen auf einer „Disposition“ beruhen, die „angeboren oder doch verhältnismäßig früh erworben“ sei und zur Verursachung einer Erkrankung im Regelfall durch äußere Reize in Form von „Kopfverletzungen“, „Alkoholismus“ oder kognitiver Überlastung ergänzt werden müsse. Seltener könnten Neurosen allein „durch von außen an das Individuum herangetragene Ursachen“ erzeugt werden [20, S. 56, S. 374, S. 384–385, S. 412].

Die Prognose gestalte sich in den seltenen Fällen, die in erster Linie auf vermeidbare äußere Faktoren zurückzuführen seien, deutlich günstiger als bei den chronisch verlaufenden, vorrangig anlagebedingten Formen [20, S. 386, S. 400].

### Neurasthenie in der zweiten Auflage

Das Konzept der Neurasthenie geht auf den US-amerikanischen Mediziner George Miller Beard (1839–1883) zurück, der in den gesellschaftlichen und technischen Neuerungen des 19. Jahrhunderts die wesentlichen Ursachen dieser Erkrankung zu erkennen glaubte [2, S. 96].

Laut Kraepelin, seit kurzem Lehrstuhlinhaber und Direktor der Psychiatrischen Universitätsklinik in Dorpat, besteht die zentrale Pathologie der Neurasthenie in einer „reizbaren Schwäche des Nervensystems“, die sowohl eine „erhöhte Erregbarkeit“ als auch eine „leichtere Ermüdbarkeit“ der Nerven zur Folge habe [20, S. 374]. Die Klinik der Neurasthenie unterteile sich in eine Grundlagensymptomatik und eine Gruppe additiver, verkomplizierender Störungsbilder – ein Schema, welches sich in den Kapiteln zu Hysterie und Epilepsie wiederholt. Die „neurasthenische Grundlage“ werde durch Freudlosigkeit, vegetative Symptome, Konzentrations‑, Gedächtnis- und die oben beschriebenen „nervösen Störungen“ bestimmt [20, S. 374–377]. Die additive Symptomatik würde sich demgegenüber durch eine „zwangsweise Ueberwältigung der ohnmächtigen psychischen Persönlichkeit durch unwiderstehlich sich ihr aufdrängende Vorstellungen, Gefühle und Impulse“ auszeichnen. Neben Zwangsvorstellungen verschiedenen Inhalts beinhalte sie auch unterschiedliche Angststörungen, allen voran die „Agoraphobie“ [20, S. 377–383].

### Hysterie in der zweiten Auflage

Die Hysterie galt immer als Frauenkrankheit, deren Ursache in der Antike in einer umherwandernden, pathogenen Gebärmutter verortet wurde, die vor allem unverheiratete, kinderlose Frauen plagen würde [38, S. 23]. Kraepelin betrachtet die schnelle und flexible Umsetzung „psychischer Zustände“ in „körperliche Reactionen“ als das eindeutigste Charakteristikum der Hysterie [20, S. 390]. Die daraus hervorgehenden nervösen Störungen sowie der „hysterische Charakter“, der bei allen schwerwiegenden Formen der Hysterie hervortrete und sich maßgeblich durch spontane Affektwechsel, eine aufbrausende, ichfixierte, exaltierte, empfindliche Art und ein starkes Bedürfnis nach Aufmerksamkeit auszeichne, würden gemeinsam die Grundlagensymptomatik der Hysterie bilden [20, S. 391–392]. Additiv könne das klinische Bild durch „hypochondrische Vorstellungen“, „depressive Verstimmungen“ oder episodische „Bewusstseinstrübungen“ verkompliziert werden [20, S. 392–397].

### Epilepsie in der zweiten Auflage

Die erste Monografie zur Epilepsie erschien im Corpus Hippocraticum. Sie deklarierte das Gehirn als Ursprungsort der Erkrankung und widersprach der damals verbreiteten Vorstellung, dass das Leiden auf eine göttliche Kraft zurückgeführt werden könne. Seine Wurzeln hat der Begriff der Epilepsie jedoch in der Vorstellung, dass eine „übernatürliche Macht“ als Krankheitsauslöser fungiere, was die „Unsicherheit, die Furcht, das Unwissen und die Machtlosigkeit“ hervorhebt, die diese Erkrankung jahrhundertelang hervorgerufen haben muss [34, S. 32–33]. Die Symptomatik der Epilepsie werde laut Kraepelin neben einer „Degeneration des Charakters“, die sich in erhöhter „gemüthlicher Reizbarkeit“, „Affectausbrüchen“, „brutalen Gewaltthätigkeiten“ und einer „Abschwächung der Intelligenz“ äußere [20, S. 405], durch episodische, meist den Krampfanfällen folgende Bewusstseinstrübungen – sog. „Dämmerzustände“ – bestimmt [20, S. 407]. Obwohl Kraepelin die Diagnose der Epilepsie in erster Instanz an das Vorhandensein von Krampfanfällen knüpft, ließ er nicht unerwähnt, dass sein psychiatrischer Kollege Paul Samt (1844–1875) auch dann eine Epilepsie annehme, wenn das klinische Bild ausschließlich von „Dämmerzuständen“ geprägt sei und Krampfanfälle gänzlich fehlen würden [20, S. 414–415].

## Dritte und vierte Auflage

In der 3. Auflage spricht sich Kraepelin auch in einem neurosenspezifischen Abschnitt zum Begriff der Funktionalität, genauer zu den „functionellen“ Störungen, aus: „ihre Localisation, ihr wechselndes Verhalten“ und der „verschlimmernde Einfluss psychischer Erregung“ gebe zu erkennen, dass ihnen keine „organischen Hirn- oder Rückenmarkserkrankungen“ zugrunde lägen [21, S. 427]. Die hier gegebene Definition „functioneller“ Störungen findet sich nicht in einer allgemeinen, krankheitsübergreifenden Einleitung zu den Neurosen, sondern in der phänomenologischen Beschreibung der „traumatischen Neurose“, einer den Neurosen nun hier 1889 neu hinzugefügten Erkrankung.

Diese ist im medizinhistorischen Sinne „zwischen … 1860 und 1920 epidemieartig über die zivilisierte Welt gegangen“, nachdem die Folgen von Eisenbahn- und Arbeitsunfällen durch die neuen Versicherungsgesetze zu einer Entschädigung berechtigten [9, S. 7]. Ihren Namen erhielt sie von Hermann Oppenheim (1857–1919), der sie in seinem 1889 erschienenen Buch zu den „traumatischen Neurosen“ als funktionelles, also morphologisch nicht greifbares, wenn auch nicht psychogenes Leiden etablierte [9, S. 32–34]. Kraepelin schließt sich postwendend den Auffassungen Oppenheims an [29, S. 123–124, S. 89–92, 21, S. 425–428] und entscheidet sich, dies unterstreichend, zunächst die Bezeichnung „traumatische Neurose“ des Berliner Kollegen zu verwenden, obwohl er den Begriff der „Schreckneurose“ eigentlich passender fände. Wenngleich er ihr noch keine rein psychogene Entstehungsweise zuweist, attestiert er ihr doch, durch „heftige Gemüthserschütterungen“ ausgelöst zu werden. Diese könnten, unter der Voraussetzung einer „individuellen Veranlagung“, bei schweren Unfällen wie „Eisenbahnkatastrophen“ auftreten. Ihre psychische Symptomatik werde durch eine „depressive Verstimmung mit ängstlichen Befürchtungen“ und „hypochondrischen Ideen“ dominiert, ihre „nervöse“ hingegen durch Schlafstörungen, Kopfdruck, Sinnesstörungen oder Obstipationen [21, S. 425–428, 22. S. 489–490].

Auch die bereits eingeführten Neurosen erleben in der 3. und 4. Auflage konzeptuelle Veränderungen. Die Neurasthenie wird, trotz gewisser Zweifel, in zwei Formen aufgeteilt: Die „erworbene“ Form, welche im Wesentlichen die Grundlagensymptomatik beinhalte, beruhe tendenziell auf äußeren Faktoren. Die „angeborene“ Neurasthenie – unter der die ehemals additiven Symptomkomplexe subsumiert werden – sei demgegenüber vorrangig auf eine „krankhafte Veranlagung“ zurückzuführen und gehe mit einer deutlich schlechteren Prognose einher [21, S. 407–411, S. 417–424].

Epilepsie und Hysterie werden um das Gedankengut der „Degenerationslehre“ ergänzt. Dieses Konzept einer generationenübergreifenden, progressiv voranschreitenden „Entartung des Menschen“ erfreute sich in der zweiten Hälfte des 19. Jahrhunderts, „gerade wegen seiner wissenschaftlichen Unschärfe“, großer Beliebtheit und geht in seiner ursprünglichen, „anthropologisch-religiösen“ Form auf Benedict Augustin Morel (1809–1873) zurück [12, 37]. Beiden Erkrankungen wird nun 1893 durch Kraepelin, mittlerweile Lehrstuhlinhaber und Klinikdirektor an der Universität Heidelberg, klar eine „Entartung“ zugrunde gelegt. Bei der Epilepsie könne sich diese auch in „Degenerationszeichen“ wie Schädelverbildungen, „Mikrozephalie, Hydrocephalus, Asymmetrien“ oder in einer „epileptischen Physiognomie“ andeuten [22, S. 491 S. 501, S. 507–508, S. 519–520]. Zusätzlich wird das Krankheitsbild der Epilepsie um die affektiven „Gleichgewichtsschwankungen“ ergänzt, deren Symptomatik maßgeblich in der Verweigerung gesellschaftlicher Pflichten bestünde: Die Kranken würden querulatorische, ängstliche oder freudig-ekstatische Stimmungszustände durchleben, während derer sie sich zurückzögen, umherirrten oder der Arbeit fernblieben [22, S. 510–511].

### Fünfte Auflage

In seiner 5., 1896 erschienenen Auflage schreibt Kraepelin einer seiner Neurosen erstmals einen psychogenen Krankheitsursprung zu: Durch ihren „Sitz“, ihre „Ausbreitung“, ihr „wechselndes Verhalten und den verschlimmernden Einfluss gemütlicher Erregung“ würden die Symptome der „Schreckneurose“ – ab der 5. Auflage wählt Kraepelin diese von ihm präferierte Bezeichnung – nicht länger ihre Funktionalität, sondern ihre psychogene Entstehungsweise offenbaren. Die „Gemüthserschütterung“ des Unfalls erzeuge eine „krankhaft erregte Einbildungskraft“, die somatische Störungen wie Anästhesien und Lähmungen an verschiedene Punkte des Körpers verlegen könne [23, S. 751–753]. Gleichzeitig verlieren die „functionell nervösen Störungen“, ohne dass dies neurosenübergreifend transparent gemacht wird, ihren bis dato seit der 2. Auflage festgehaltenen obligaten Charakter. So geht Kraepelin nun in Analogie zu Samt auch in solchen Fällen von einer Epilepsie aus, deren klinisches Bild keinerlei Krampfanfälle aufweisen würde [23, S. 724]. Gleichzeitig erhalten die Neurosen in Form episodischer Störungen ein neues krankheitsübergreifendes Charakteristikum [23, S. 702].

Die augenfälligste, krankheitsspezifische Veränderung der 5. Auflage besteht darin, dass die Neurasthenie in drei eigenständige Krankheitseinheiten zerfällt, die allesamt nicht länger den Neurosen zugerechnet werden. Der „chronisch nervösen Erschöpfung“ fallen all jene Symptome zu, die zuvor der „erworbenen“ Neurasthenie zugeordnet wurden, wohingegen die Krankheitszeichen der „angeborenen Neurasthenie“ unter der „constitutionellen Verstimmung“ und dem „Zwangsirresein“ aufgeteilt werden. Die Abspaltung der „chronisch nervösen Erschöpfung“ erfolgt dabei anhand der bereits in Auflage 3 etablierten symptomatischen und prognostischen Trennlinien. Ihre Symptome könnten „auch bei gesunder Veranlagung durch dauernd einwirkende erschöpfende Ursachen erzeugt werden“ und könnten, so folgt Kraepelin dem Leipziger Nervenarzt Paul Julius Möbius (1853–1907), als Zeichen einer „chronischen Vergiftung durch Ermüdungsstoffe“ betrachtet werden [23, S. 341–342, S. 347, S. 757–774].

### Sechste Auflage

Die 6. Auflage ist durch eine Weiterentwicklung der einzelnen, jeweils eine konkrete Neurose betreffenden ätiologischen Theorien geprägt.

Im Falle der Schreckneurose hegt Kraepelin in seiner 6. Auflage von 1899, also nur drei Jahre später, offene Zweifel an der dominierenden ätiologischen Bedeutung der „Gemütsbewegungen“. Sie würde „unter dem Einflusse des Unfallversicherungsgesetzes nicht nur rasch an Häufigkeit“ zunehmen, sondern auch ungünstiger verlaufen. Entsprechend würde er ihre entscheidendste Ursache nun hinter psychosozialen, also der Wechselwirkung zwischen psychischen und sozialen Prozessen geschuldeten, Mechanismen vermuten, die das Rentenverfahren mit sich bringe [24; II, S. 524–525].

Das eindeutigste Charakteristikum der Hysterie, das Kraepelin in den Jahren davor als schnelle und flexible Umsetzung „psychischer Zustände“ in „körperliche Störungen“ verstand [24; II, S. 492], erfährt in der 6. Auflage eine Konkretisierung. Der Lehrbuchautor erklärt nun, und schließt sich damit Möbius’ Ansichten an, dass es sich bei den „psychischen Zuständen“ um „gefühlsstarke Vorstellungen“ handele. In Abgrenzung zu Möbius mache er diese jedoch auch für die psychischen Krankheitszeichen der Hysterie verantwortlich [24, II, S. 510–512]. Zusätzlich gewinnt die psychische Symptomkomponente im Gesamtbild der Hysterie stark an Gewicht, da Kraepelin es nun als „schweren Fehler“ betrachtet, die charakterliche „Entartung“ der Hysterie nicht als unabdingbaren Teil des klinischen Bildes anzusehen [24, II, S. 502].

Das Substrat der Epilepsie stuft er als Gegenstand anhaltender wissenschaftlicher Debatten ein: Sowohl die histopathologische als auch die biochemische Forschung liefere zahlreiche Thesen zu ihrer körperlichen Grundlage. So könnten epileptische Anfälle als Reaktion auf periodisch anflutende, körpereigene Gifte verstanden werden. Er selbst stünde der Bestrebung, die Epilepsie als Stoffwechselerkrankung aufzufassen, aufgrund ihrer „Erblichkeitsbeziehung … zu anderen Geistes- und Nervenkrankheiten“ jedoch noch skeptisch gegenüber und lokalisiere die zentrale Pathologie der Epilepsie weiterhin in den „Zuständen des Nervengewebes“ [24, II, S. 479–482].

### Siebte Auflage

In der 7. Auflage bricht der seit Herbst 1903 schweren Herzens nach München an die dortige Universitätsnervenklinik übergewechselte Lehrbuchautor mit seiner gesamten bisherigen Neurosenkonzeption: Neurosen anhand ihrer „nervösen Störungen“, die für ihn, wie dargestellt, schon ab der 5. Auflage an Relevanz verloren haben, von den Psychosen zu unterscheiden, empfinde er fortan als „unhaltbar“. Weder gebe es „Psychosen ohne nervöse Begleiterscheinungen noch Neurosen ohne psychische Störungen“. Er halte es jedoch für sinnvoll, einen kleinen Formenkreis aus der bisherigen Gruppe der Neurosen beizubehalten, welcher sich durch eine rein „psychogene Entstehungsweise der einzelnen Krankheitserscheinungen“ auszeichnen würde [25, II, S. 683].

Diesen „psychogenen Neurosen“ fügt er, neben der Schreckneurose und der Hysterie, noch das Krankheitsbild der „Erwartungsneurose“ hinzu, deren Symptome einer „ängstlichen Erwartung“ entsprängen und in schmerzhaften Körperempfindungen, Lähmungen oder Krämpfen bestünden, die den Vollzug gewöhnlicher Tätigkeiten wie Lesen, Schlucken oder Gehen verhindern würden [25, II, S. 731–737].

### Achte Auflage

In der 8., zwischen 1909 und 1915 erschienenen, auf 3048 Seiten in 4 Bänden angewachsenen Auflage findet sich nun als Endpunkt der Nosologieentwicklung Kraepelins anstelle der „psychogenen Neurosen“ die Oberkategorie der „psychogenen Erkrankungen“. Diese wird um das „induzierte Irresein“, den „Verfolgungswahn der Schwerhörigen“, die „psychogenen Geistesstörungen der Gefangenen“, den „Querulantenwahn“ sowie die „nervöse Erschöpfung“ erweitert, die bis zur 4. Auflage als Symptomkomplex der Neurasthenie bereits Teil der ursprünglichen Neurosenkategorie gewesen ist. Die hier einsortierten Erkrankungen würden sich durch die „psychogene Entstehungsweise“ ihrer Symptome auszeichnen und könnten auf „Schicksalsschläge“, „Tätigkeiten“ oder besondere Lebensbedingungen zurückgeführt werden [26, IV, S. 1397–1399].

**Abb. 2:**
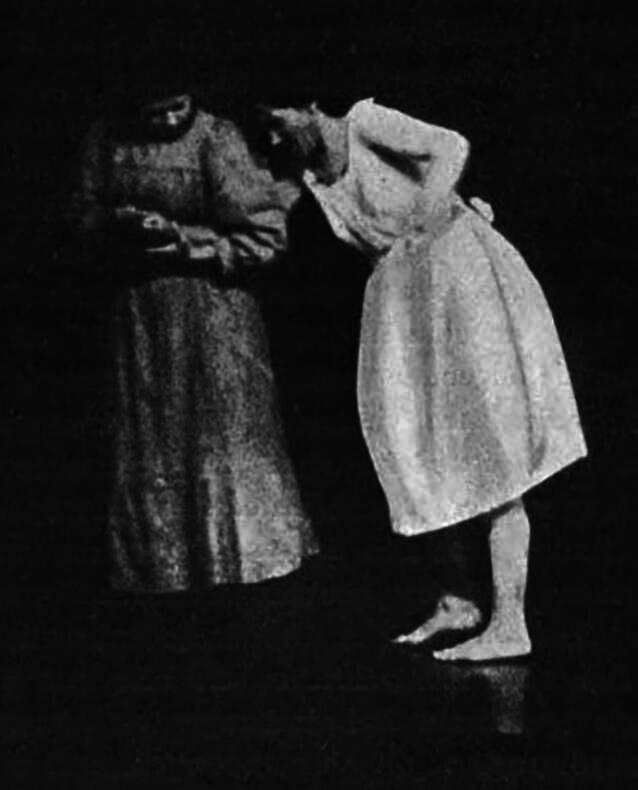
Gehstörungen bei „traumatischer Neurose“ [26, S. 1474]

Die Hysterie wird dieser Gruppe enthoben und von Kraepelin nun als „anlagebedingte psychische Entwicklungshemmung“ verstanden, die zu „atavistischen“, menschheitsgeschichtlich überholten „Reaktionen auf emotionale Reize führt“ [37, S. 50]. Im Gegensatz dazu verbleibt die „Schreckneurose“ unter den „psychogenen Erkrankungen“, zerfällt jedoch in zwei unabhängige Krankheitsentitäten, deren Konzeption sich an den in der 6. Auflage formulierten Zweifeln zu ihrer Ätiologie orientiert. Die „traumatische Neurose“, der die Mehrzahl aller ehemals unter der Schreckneurose subsumierten Fälle zufalle, könne auf den „Einfluß, den die Aussicht auf Entschädigung im Seelenleben des Unfallverletzten ausübt“, zurückgeführt werden und weise einen „schleppenden“ Verlauf auf [26, IV, S. 1476; S. 1484]. Demgegenüber könne die „Schreckneurose“, ähnlich der Hysterie, als Ausdruck „stammesgeschichtlich alter“ Schutz- und Abwehrstrategien verstanden werden. Diese träten bei Konfrontation mit erschreckenden Bewusstseinsinhalten bei denjenigen Individuen zum Vorschein, bei welchen die Verarbeitungsstrategien einer höheren menschlichen „Entwicklungsstufe“ ungenügend ausgebildet seien [26, IV, S. 1451–1452]. Die 8. Auflage enthält zudem zwei Fotografien zur Veranschaulichung der Symptomatik der „traumatischen Neurose“. Eine davon wird exemplarisch in Abb. 2 präsentiert.

## Diskussion

### Die modernen Nachfolger der intitialen Neurosen Kraepelins

Nur ein Teilkonzept seiner initialen Neurosen „Hysterie“, „Epilepsie“ und „Neurasthenie“ wird am Endpunkt der Nosologieentwicklung Kraepelins noch unter den „psychogenen Erkrankungen“ aufgeführt, die als das Nachfolgekonzept der ursprünglichen Neurosenkategorie Kraepelins verstanden werden können. Hierbei handelt es sich um die „nervöse Erschöpfung“, deren Symptomatik ein Teilgebiet der ursprünglichen Neurasthenie abdeckt [26, IV, S. 1400–1416].

In Analogie dazu wurde der Begriff der Neurasthenie, der in den 1980er-Jahren wieder stärker in den Fokus der westlichen Medizin rückte [32], noch in der erst seit dem Jahr 2022 abgelösten Klassifikation psychischer Erkrankungen des ICD-10 unter der Kategorie „andere neurotische Störungen“ geführt [4] – ganz im Gegensatz zu den Nachfolgeerkrankungen der „Hysterie“ und „Epilepsie“. Das wesentlichste obligatorische Kriterium dieses modernen Neurastheniebegriffes, das anhaltende Erschöpfungsgefühl nach geringfügiger geistiger oder körperlicher Anstrengung (ICD-10), erinnert eindeutig an das Kernsymptom der „Ermüdung“ in Kraepelins „nervöser Erschöpfung“ [26, S. 1400–1416]. Im Unterschied zu der modernen Neurastheniekonzeption tritt die „nervöse Erschöpfung“ jedoch in Folge einer „übermäßigen“ oder „zu lange fortgesetzten geistigen oder körperlichen Arbeit“ auf [26, S. 1400]. Das ICD-11 listet den Begriff der Neurasthenie nicht mehr als eigenständige Diagnose auf und integriert die unter ihm klassifizierten Symptome in die neue Kategorie der „bodily distress disorder“ [30]. Parallel zu diesen psychiatrischen Krankheitsentitäten können das „Chronic-Fatigue-Syndrom“ (CFS), die „Fibromyalgie“ (FM) und die „multiple chemische Sensitivität“ (MCS) als das „organische Erbe“ des ursprünglichen Neurastheniekonzeptes aufgefasst werden [33].

Im Unterschied zur Neurasthenie gelten Epilepsien heutzutage nicht mehr als Neurosen, sondern als heterogene Gruppe neurologischer Erkrankungen, deren Gemeinsamkeit in einer pathologischen elektrischen Aktivität der gehirneigenen Nervenzellen besteht [1]. Die Begriffsgeschichte der Hysterie setzte sich abseits psychoanalytischer Betrachtungsweisen, in denen die Bezeichnung bis heute Verwendung findet [5, 27, 31], im ICD-10 in dem Begriff der „histrionischen Persönlichkeitsstörung“ fort, die die Kategorie der „hysterischen Persönlichkeit“ ersetzt hat [10]. Mit Ausnahme eines unangemessen verführerischen Verhaltens lassen sich die störungsspezifischen Kriterien der „histrionischen Persönlichkeitsstörungen“ sämtlich in Kraepelins Beschreibungen des „hysterischen Charakters“ wiederfinden [25, S. 684–S. 695].

### Die Einflussfaktoren der Evolution der Neurosenkonzeption Kraepelins

Anhand der Evolution der Konzeption Kraepelins lässt sich exemplarisch das Aufkommen eines neuen Verständnisses der Neurosen nachvollziehen: Sie wandeln sich von funktionellen, das Nervensystem betreffenden Störungen zu Erkrankungen psychogenen Ursprungs. Für diese hier summarisch referierte, etwa über 30 Jahre währende Entwicklung lässt sich ein breites Spektrum an sowohl gesellschaftlichen als auch fachlich nervenärztlichen Einflussfaktoren identifizieren.

Im Vergleich mit den anderen Neurosen Kraepelins bildete allen voran die Schreck- oder traumatische Neurose einen wichtigen Motor für die Entwicklung seiner Neurosenkonzeption. Sie war die erste neurotische Erkrankung, der durch Kraepelin dezidiert eine psychogene und, nach ihrer Aufspaltung in der 8. Auflage, eine psychosoziale Entstehungsweise zugeschrieben wurde [22, S. 489, 26, IV, S. 1451, 21, S. 427]. In seiner 3. Lehrbuchauflage, die kurz nach Oppenheims durchschlagskräftiger Monografie von 1889 erschien, schloss sich Kraepelin zunächst weitestgehend den Auffassungen Oppenheims an [29, S. 123–124, S. 89–92; 21, S. 425–428]. Oppenheims funktionelles Verständnis der traumatischen Neurose stieß allerdings schon kurz darauf, auf dem internationalen Berliner Ärztekongress von 1890, auf Gegenwind. Seine Kritiker argwöhnten simulierende Patienten oder machten die Gegebenheiten der Unfallversicherung oder Suggestionswirkungen für die traumatische Neurose verantwortlich [16]. Im Mittelpunkt der Diskussion stand die Frage, inwiefern die Erkrankung als bloßer Simulationsversuch aufgefasst werden könne. Dies hebt hervor, dass die Ärzteschaft, durch den aus der Erkrankung hervorgehenden Entschädigungsanspruch, in die besondere Situation geriet, in ihrem Handeln nicht nur den Patienten, sondern auch der Reichsökonomie verpflichtet zu sein [9]. Kraepelin schrieb der traumatischen Neurose jedoch zunächst 1896 eine psychogene Entstehungsweise zu, bevor er sich ab der 6. Auflage allmählich der Idee einer psychosozialen Bedingtheit des Leidens öffnete [24, II, S. 524–525]. Seine kurze, 1896 formulierte Erklärung zur Psychogenie der Schreckneurose [23, S. 751–753] erinnert an das Modell zur Entstehung „traumatischer Hysterien“ des berühmten französischen Neurologen Jean Martin Charcot (1825–1893). Laut diesem niste sich die Idee, dass ein Körperteil ernsthaft verletzt sein könne, im Moment des Schocks parasitär in das „unterbewusste Vorstellungsleben“ der Patienten ein und wachse dort heran, bis sie tatsächliche, physische Symptome hervorrufen würde [8]. Kraepelins einstweilige Hinwendung zu diesem Konzept kann vielleicht damit erklärt werden, dass er sich – wie Charcot selbst [8] – aufgrund seiner materialistisch-naturwissenschaftlichen Denkweise einem Bild, welches der Lehre von Krankheitserregern entlehnt war, zunächst besser annähern konnte als einem psychosozialen Erklärungsversuch.

Besonders in der 5. und 6. Auflage von 1896 bzw. 1899 wurde Möbius deutlich zum Referenzpunkt Kraepelins, vor allem hinsichtlich ätiologischer und nosologischer Überzeugungen. Jetzt wird deutlich, dass diese Feststellung [36] auch für seine Konzeption der Neurosen gilt. Kraepelins Leipziger Kollege und Freund hatte unter dem Eindruck der Degenerationslehre zwischen 1890 und 1893 für die Nerven- und psychischen Erkrankungen Klassifikationen entworfen, die allein auf dem Kriterium der Krankheitsursache basierten. Bei den exogenen Erkrankungen sah Möbius auf das Nervensystem einwirkende Gifte wirken. Kraepelin übernahm diese Auffassung in der 5. Auflage für seinen Neuentwurf der Neuasthenien [23, S. 347]. Zusätzlich schloss er sich in der 6. Auflage Möbius’ Sichtweise an, die körperlichen Symptome der Hysterie seien Folgeerscheinungen „gefühlsstarker Vorstellungen“ [24, II, S. 510–512].

Methodisch hat Kraepelin auch bei der Entwicklung seiner Neurosenkonzeption auf das von ihm um 1890 inaugurierte Forschungsprinzip der empirisch-klinischen Psychiatrie zurückgegriffen. Dafür waren Vorarbeiten Karl Ludwig Kahlbaums (1828–1899) konstitutionell, die Kraepelin zu Beginn seiner Dorpater Zeit als bahnbrechend erkannte. Nicht nur, dass er Kahlbaums Nosologie in den 4. bis 6. Auflagen seines eigenen Lehrbuchs zu einem ganzen System psychischer Erkrankungen weiterentwickelte, sondern er entlehnte Kahlbaums Forderung der Verlaufsbeobachtung der Krankheiten als wohl wesentlichsten Parameter seiner empirisch-klinischen Forschungsmethodik, auf der sein eigenes klassifikatorisches Schaffen basierte [17, 35, 39]. Diese Methodik hat seine Aufspaltung der „Neurasthenie“ motiviert, aber auch seine Auffassung von den „Unfallneurosen“ beeinflusst. So fällt bei der Umgestaltung der Neurasthenie in der 5. Auflage, die die erste klinische, an „Entstehungsbedingungen,… Verlauf und … Ausgang“ orientierte Klassifikation psychischer Erkrankungen darbot und sich nicht vorrangig auf symptomatische Beobachtungen stützte [3, S. 24], die Erkenntnis ins Gewicht, dass ihre „angeborene“ Form mit einer deutlich ungünstigeren Prognose einhergehe [21, S. 417, 23, S. 348, S. 773]. Auch seine ab der 6. Auflage ins Feld geführten Zweifel an dem ätiologischen Konzept der „Schreckneurose“, die schließlich in ihrer Neugruppierung mündeten, begründete Kraepelin mit der Beobachtung, dass die Erkrankung unter dem Einfluss des Unfallversicherungsgesetzes merklich ungünstiger verlaufen würde [21, S. 417, 24, II, S. 524].

Bei dem Aufbau seiner berühmten Nosologie ließ sich Kraepelin, wie bereits die krankheitskonzeptionellen Anstöße seiner Kollegen verdeutlicht haben, neben dem Krankheitsverlauf und der Symptomatik noch von weiteren Parametern leiten. Wie Hoff gezeigt hat, unterschied sich der von Kraepelin in der 2. bis 8. Lehrbuchauflage vertretene Krankheitsbegriff wesentlich von den Vorstellungen seiner programmatischen Arbeiten, die von 1918 bis 1920 erschienen sind [13]. So ging er in seinem Lehrbuch von der Existenz naturgegebener Krankheitsentitäten aus, die jeweils auf ätiologischer, symptomatischer und pathoanatomischer Ebene zum Ausdruck kämen und sich, gesetzt den Fall, dass man auf einem dieser Gebiete eine „erschöpfende Kenntnis aller Einzelheiten“ erreicht hätte, theoretisch nur mithilfe dieser einen Forschungsmethode kategorisieren lassen müssten [13, 23, S. 314]. Er resümierte, dass sich eine Einteilung psychischer Erkrankungen, eben aufgrund einer jeweils unvollkommenen Kenntnis der verschiedenen Forschungsgebiete, auf das Zusammenspiel ätiologischer, symptomatischer, pathoanatomischer und zusätzlich prognostischer Forschungsergebnisse stützen müsse [21, S. 238]. Im Vergleich dazu sind die einzelnen diagnostischen Gruppen in der aktuellen ICD-11 anhand mutmaßlicher gemeinsamer ätiologischer und pathophysiologischer Faktoren, übereinstimmender phänomenologischer Parameter und einer Entwicklungsperspektive geordnet [30]. Die in seinen Lehrbüchern artikulierte nosologische Haltung Kraepelins, die stets mehrere Anschauungsprinzipien berücksichtigte, mag auch die Evolution seiner Neurosenkategorie stark beeinflusst haben. So musste der Neurosenbegriff mit dem Ausbau der ätiologischen Konzepte ab der 6. Auflage stark divergierende Ursachentheorien vereinen. Vielleicht haben die biochemischen Erklärungsmodelle der Epilepsie, in Gegenüberstellung mit den psychosozialen und gesundheitspolitischen Ursachen der Schreckneurose, das Fassungsvermögen einer gemeinsamen Krankheitskategorie überschritten [24, II, S. 479–482, S. 524–525] und haben auf diesem Wege zur Auflösung der ursprünglichen Neurosenkonzeption in der 7. Auflage beigetragen.

In seinen späteren, ab 1918 verfassten Arbeiten entwickelte Kraepelin einen differenzierteren, methodenkritischen Krankheitsbegriff und plädierte dafür, dass anhand psychischer Symptome nicht direkt auf physische Krankheitsvorgänge rückgeschlossen werden könne. Er spekulierte, dass die üblichen klinischen Einheiten keine „direkten Äußerungsformen der zugrundeliegenden … Krankheitsvorgänge“ seien, sondern erst im Zusammenspiel mit „sekundären Faktoren wie Primärpersönlichkeit, psychosoziales Umfeld etc“ [13, S. 512] entstehen würden. Mit dieser Theorie näherte er sich der Meinung seines Kritikers Alfred Hoche (1865–1943) an, der von der Vorstellung von Krankheitsentitäten abrückte und von Symptomkomplexen ausgehend klassifizieren wollte [3, S. 33–24]. Ansätze dieser späteren Auffassung lassen sich in den zunehmend komplexer gestalteten Krankheitsmodellen der 8. Auflage erkennen. In seinen frühen Lehrbuchauflagen hatte sich Kraepelin größtenteils darauf beschränkt, das Entstehen der Neurosen sehr allgemein durch das Zusammenspiel äußerer Faktoren mit einer inneren Veranlagung zu erklären [20, S. 374, 22, S. 467]. In seiner 8. Lehrbuchauflage wurde dagegen gerade die Darstellung jener Einflussfaktoren, die er unter dem Begriff der „Disposition“ zusammengefasst hatte [20, S. 374], merklich erweitert. So verstand er die Klinik seiner nun von der „traumatischen Neurose“ zu unterscheidenden „Schreckneurose“ als Ausdruck „stammesgeschichtlich alter“ Schutz- und Abwehrstrategien, die als Reaktion auf erschreckende Bewusstseinsinhalte jedoch nur bei denjenigen Individuen zum Vorschein träten, bei welchen die Verarbeitungsstrategien einer höheren, menschlichen „Entwicklungsstufe“ ungenügend ausgebildet seien [26, IV, S. 1451–52].

Auch der Berücksichtigung der Degenerationslehre kam zweifellos eine zunehmende und schließlich weitreichende Bedeutung für die Entwicklung des Neurosenkonzeptes Kraepelins zu. Dies passt zu Hoffs Befund, dass kein Zweifel bestehe, dass Kraepelin die Degenerationstheorie akzeptierte und in die Debatte über die Ätiologie und Pathogenese psychischer Störungen einbrachte [15]. So kann ihr ein deutlicher Einfluss auf ein wichtiges Neurosencharakteristikum nachgewiesen werden, dessen Bedeutungszuwachs entscheidend zu Kraepelins Abkehr von seiner ursprünglichen Neurosenkonzeption beigetragen hat: die psychische Symptomkomponente. In der 1. Auflage als regelmäßiger, jedoch nicht explizit obligatorischer Bestandteil eingestuft [19, S. 22], vollzog sich ihr eindeutigster Bedeutungszuwachs in der Hysteriekonzeption der 6. Auflage. Wie beschrieben, bezeichnete Kraepelin es hier als „schweren Fehler“, die „Entartung“ – synonym für die hysterischen Charakterveränderungen verwendet – nicht als obligaten Teil des Krankheitsbildes zu betrachten [24, II, S. 502]. Scheinbar daran anknüpfend, begründete er die konzeptuelle Neuordnung der 7. Auflage mit dem obsoleten Charakter der Vorstellung, Neurosen würden sich in Abgrenzung zu den Psychosen durch ein körperlich-nervöses Symptomspektrum auszeichnen [25, II, S. 683]. Indem also die Degenerationslehre, die für ihn mit der Idee eines grundsätzlich „aus der Art geschlagenen Menschen“ verknüpft war [24, II, S. 95], stärkere Berücksichtigung in seiner Neurosenkonzeption fand, verblasste gleichzeitig die Trennschärfe zwischen den Neurosen und anderen psychischen Erkrankungen.

Darüber hinaus kann das Ende der ursprünglichen Neurosenkonzeption Kraepelins als Ausdruck einer zunehmenden ärztlichen und zivilgesellschaftlichen Offenheit gegenüber psychogenen Krankheitsentwürfen verstanden werden. Vielleicht verschwand um und nach der Jahrhundertwende das Gebot, Erkrankungen ohne pathoanatomisches Korrelat mithilfe einer unentdeckten, jedoch klar auf körperlicher Ebene verorteten Läsion des Nervensystems zu legitimieren. Das nur empirisch-naturwissenschaftliches Schlussfolgern akzeptierende Zeitalter war dabei, seinen Zenit zu überschreiten, monokausale Ableitungen wurden durchbrochen. Ärzte wie Piere Janet (1859–1947), Charcot, Möbius, Freud, Josef Breuer (1842–1925) und Arthur Schnitzler (1862–1931) gewannen mit ihrer Auffassung zum psychogenen Ursprung psychischer Krankheiten mehr und mehr Einfluss in den Intellektuellenkreisen und damit auch unter der Kollegenschaft.

## Fazit

- Anhand der Entwicklung der Neurosenkonzeption Kraepelins lässt sich exemplarisch der Wandel der Neurosen zu psychogenen Erkrankungen nachvollziehen.
- Die „traumatische Neurose“ bildete einen wichtigen Motor für die Entwicklung der Neurosenkonzeption Kraepelins.
- Möbius’ krankheitskonzeptionelle Auffassungen waren auch im Bereich der Neurosen eine wichtige Inspirationsquelle Kraepelins.
- Die stärkere Berücksichtigung der Degenerationslehre hat entscheidend zur Auflösung der ursprünglichen Neurosenkategorie Kraepelins beigetragen.
- Kraepelins empirisch-klinische Forschungsweise, die besonderes Augenmerk auf Verlaufsbeobachtungen legte, hat auch die Entwicklung seiner Neurosenkonzeption entscheidend geprägt.
- Ansätze des späten, methodenkritischen Krankheitsbegriffs Kraepelins lassen sich in den komplexer gestalteten Krankheitsmodellen der 8. Auflage, insbesondere anhand der Krankheitskonzeption der „Schreckneurose“, erkennen.
